# Oncogenic miR-9 is a target of erlotinib in NSCLCs

**DOI:** 10.1038/srep17031

**Published:** 2015-11-23

**Authors:** Xi Chen, Lingjun Zhu, Zhuo Ma, Geng Sun, Xuan Luo, Min Li, Sulan Zhai, Ping Li, Xuerong Wang

**Affiliations:** 1 Department of Pharmacology, Nanjing Medical University, Nanjing, Jiangsu Province, China, 210029; 2 Department of Oncology, the First Affiliated Hospital of Nanjing Medical University, Nanjing, Jiangsu Province, China, 210029

## Abstract

EGFR-targeted cancer therapy is a breakthrough in non-small cell carcinoma. miRNAs have been proved to play important roles in cancer. Currently, for the role of miRNAs in EGFR-targeted cancer therapy is unclear. In this study, first we found that erlotinib reduced the expression of miR-9. MiR-9 expression was increased in human lung cancer tissues compared with peripheral normal tissues, and miR-9 promoted the growth of NSCLC cells. Overexpression of miR-9 decreased the growth inhibitory effect of erlotinib. Second, miR-9 decreased FoxO1 expression by directly inhibition of its mRNA translation. Adenovirus-mediated overexpression of FoxO1 or siRNA-mediated downregulation of FoxO1 negatively regulated cell growth. And exogenous overexpression FoxO1 reduced the pro-growth effect of miR-9. Finally, we found that erlotinib upregulated FoxO1 protein expression. Moreover, overexpression of miR-9 decreased erlotinib-induced FoxO1 expression, and overexpression of FoxO1 enhanced the growth inhibitory effects of erlotinib. Additionally, we found that erlotinib downregulates miR-9 expression through suppressing the transcrption of miR-9-1 and enhanced DNA methylation maybe involved. These findings suggest that oncogenic miR-9 targeted FoxO1 to promote cell growth, and downregulation of this axis was involved in erlotinib’s growth inhibitory effects. Clarifying the regulation of miRNAs by erlotinib may indicate novel strategies for enhancing EGFR-targeted cancer therapy.

Lung cancer is the leading cause of cancer related deaths. It has one of the lowest survival rates among all cancers with a 5-year survival rate of 16%^1^. The non-small cell lung carcinoma (NSCLCs) accounts for about 85% of lung cancers^2^. For the reason that most of the patients were diagnosed at late stage, chemotherapy and molecular targeted cancer therapy were commonly used either solely or in combination with surgery and radiotherapy^3^. Aberrant activity or overexpression of epidermal growth factor receptor (EGFR) plays a critical role in NSCLCs, and targeting EGFR is a breakthrough in lung cancer treatment^4^. EGFR tyrosine kinase inhibitors (EGFR-TKIs), such as erlotinib or gefitinib, mainly function through blocking the ATP-binding pocket of the EGFR and suppressing two major signaling pathways in cancer - PI3K/Akt and Ras/MAPK pathway^5^. Even though these small molecular inhibitors are very effective for a subgroup of patients including those with EGFR active mutations, currently its outcome is quite limited for the majority of lung cancer patients^6^. To enhance EGFR-targeted cancer therapy, better understanding of the mechanisms and consequences of EGFR inhibition other than blocking EGFR are urgently needed.

microRNAs (miRNAs) are 18–22 nt, small, and non-coding RNAs, that negatively regulate gene expression at the post-transcriptional level by directly binding with the 3′ untranslated regions (3′ UTR) of target mRNAs to induce mRNA degradation or suppress mRNA translation^7^. Many miRNAs have been proved to play critical roles in cell growth, differentiation, apoptosis, motility, and drug resistance, and are involved in several types of diseases including cancer^8^. miRNA expression patterns in cancer are tissue- and cell type- dependent. MiR-9 has been shown to regulate growth, differentiation, migration, and apoptosis of cancer cells, either as an oncogene or as a tumor suppressor depending on different cancer types^9^. Even though Gomez *et al.* just reported that miR-9 was involved in EGFR signaling pathway, for its role and mechanism in lung cancer with EGFR inhibition was still unknown.

FoxO1 is a member of the forkhead box (Fox) O transcription factor family. It is a key effecter of Akt and SGK1 signaling pathway, and regulates cell cycle arrest, energy metabolism, DNA repair, oxidative stress resistance, and apoptosis^10^. Reduced expression of FoxO1 is detected in several types of cancers, such as endometrial cancer and lung cancer, suggesting it is a tumor suppressor^11^,^12^. When FoxO1 is phosphorylated by some kinases, such as Akt, it is sequestered in the cytoplasm and degradated through ubiquitination pathway, thereby blocking its nuclear localization and decreasing its target genes transcription^13^. Recently, it was identified to be a target of several miRNAs, such as miR-27a and miR-9^11^. However, whether its regulation by erlotinib involves miRNAs is unclear.

In this study, we first determined the oncogenic role of miR-9 by detecting the expression of miR-9 in human lung cancer tissues and the effect of miR-9 on the growth of NSCLC cells. We then detected the effects of miR-9 on FoxO1 expression levels. Finally, we tested the effects of erlotinib on FoxO1 expression through miR-9. In addition, we examined how erlotinib regulated miR-9 expression. Our study clarifies a new mechanism of erlotinib through regulation of miR-9 - Foxo1 in lung cancer and suggests targeting miR-9 to enhance the anticancer efficacy of erlotinib.

## Results

### MiR-9 is a target of erlotinib in NSCLC cells

**Figure 1:**
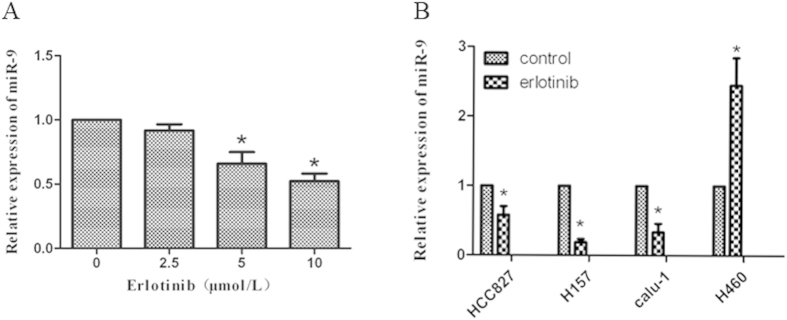
Erlotinib downregulated miR-9 expression. (**A**) A549 cells were treated with erlotinib 2.5, 5, 10 μmol/L for 72 h. (**B**) HCC827 cells were treated with erlotinib 50 nmol/L, and Calu-1, H157, H460 cells were treated with erlotinib 5 μmol/L for 24 h. The total RNAs were purified and subjected to qRT-PCR assay. Columns, means of three replicate determinations; bars, SD. *P < 0.05.

Erlotinib is an EGFR inhibitor, currently for its effects on miRNAs are still unclear. To understand the mechanism of erlotinib other than EGFR inhibition in NSCLCs, we investigate its effect on miR-9 expression. Figure 1A showed that erlotinib decreased miR-9 levels in a dose-dependent manner in A549 cells, which contains wild type EGFR. Erlotinib also decreased miR-9 expression in HCC827 (with mutant EGFR), H157, and Calu-1 cells, but not in H460 cells (Fig. 1B). It suggests that miR-9 is regulated by erlotinib in NSCLCs.

**Figure 2:**
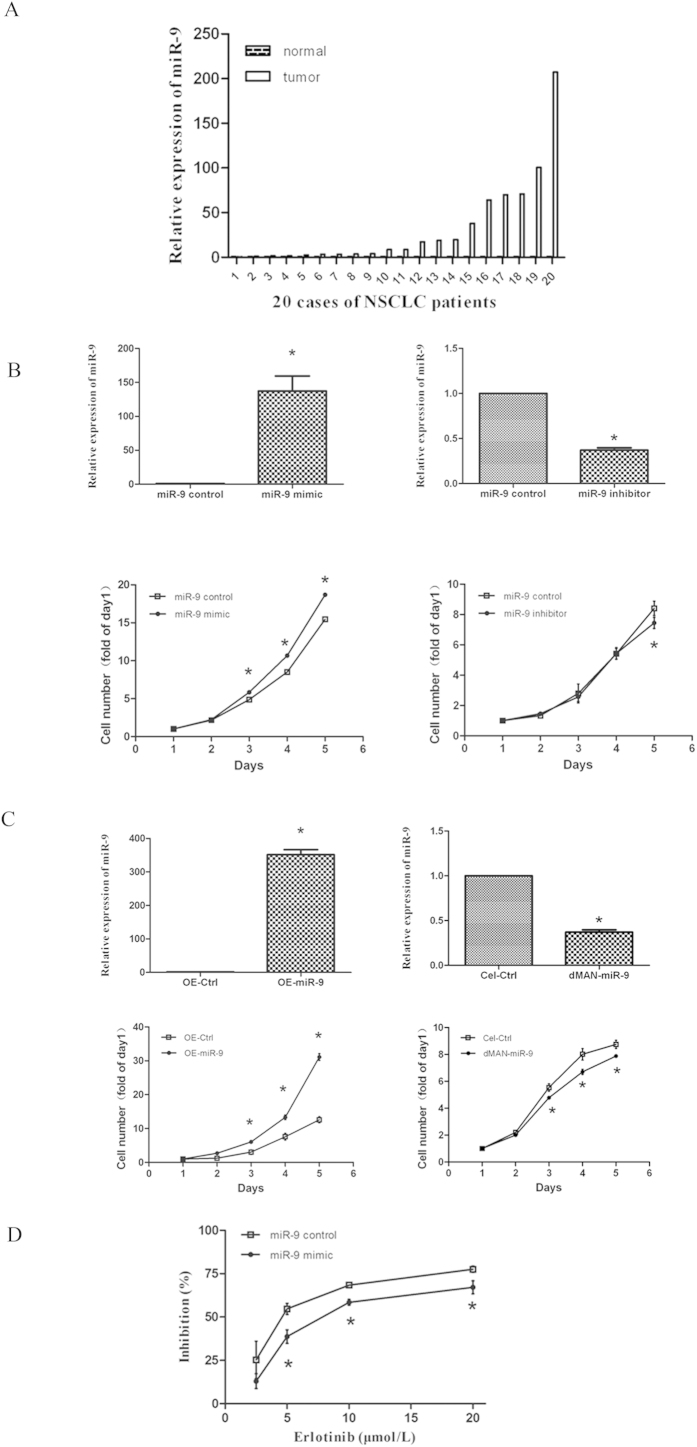
MiR-9 is oncogenic in NSCLCs and overexpression of miR-9 reduced the growth inhibitory effect of erlotinib. (**A**) the expression of miR-9 was elevated in human lung cancer tissues compared with the paired peripheral normal tissues. Total-RNA was purified from human tissue samples of 20 lung cancer patients and subjected to qRT-PCR assay. (**B**) A549 cells were transfected with synthetic miR-9 mimic (left) or inhibitor (right), or their relative control, and subjected to qRT-PCR assay (up) or a 5-day SRB assay (low). (**C**) A549 stable cell lines with increased (OE-miR-9/OE-Ctrl) (left) or decreased (dMAN-miR-9/Cel-Ctrl) (right) miR-9 expression were subjected to qRT-PCR assay (up) or a 5-day SRB assay (low). (**D**) A549 cells were transfected with synthetic miR-9 mimic and its control for 24 h, then reseeded to 96-well plates. On the second day, cells were treated with different concentrations of erlotinib for another 72 h and subjected to SRB assay. Relative expression of miR-9 was calculated using the 2^−ΔΔCt^ method. Columns, means of three replicate determinations; points, means of four replicate determinations; bars, SD. *P < 0.05. The data are representatives of three independent experiments.

miR-9 has been identified as both an oncogene and a tumor suppressor depending on different cancer types. To clarify its role in lung cancer, we first detected its expression in human lung cancer tissues. miR-9 expression was examined in lung cancer tissues and their matched peripheral normal tissues of 20 NSCLC patients using qRT-PCR assay. 19/20 cases showed significant elevated miR-9 expression compared with the normal tissues. And only one case showed decreased miR-9 expression in cancer tissues (Fig. 2A). Among these 19 cases, 15 cases showed more than 3 folds increase of miR-9. These results suggest that miR-9 maybe oncogenic in NSCLCs.

We next determined the effects of manipulating miR-9 expression on the growth of NSCLC cells. Figure 2B showed that transiently transfection of miR-9 mimic, or its inhibitor successfully increased or decreased miR-9 expression levels in A549 cells, respectively. Furthermore, exogenous overexpression of miR-9 promoted cells growth significantly, whereas inhibition of endogenous miR-9 expression inhibited cell growth, even though slightly (Fig. 2B). To confirm the pro-growth effect of miR-9, we established stable cell lines with miR-9 overexpression and its control by infection with lentivirus OE-miR-9 and OE-ctrl, respectively. In parallel, stable cell lines with miR-9 downregulation were established by dMAN-miR-9 lentivirus and its control Cel-ctrl. As shown in Fig. 2C, stable cell lines with successful upregulation of miR-9 expression promoted cell growth significantly, whereas stable cell lines with miR-9 suppression inhibited cell growth. These results suggest that miR-9 functions as an oncogene in lung cancer.

To identify the role of miR-9 in erlotinib induced cell growth inhibition, we transiently transfected miR-9 mimic in A549 cells and then treated the cells with different concentrations of erlotinib. Figure 2D showed that miR-9 mimic partially abrogated the inhibitory effects of erlotinib on cell growth. In summary, these results suggest that downregulation of oncogenic miR-9 expression play an important role in mediating the anticancer effect of erlotinib.

### FoxO1 is a target of miR-9

**Figure 3:**
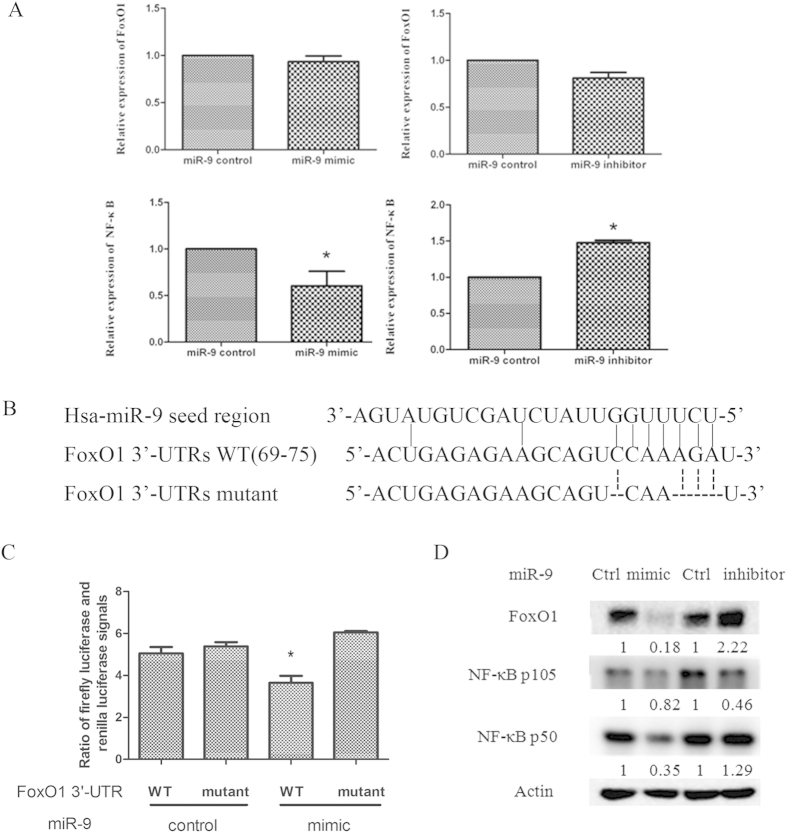
FoxO1 is a target of miR-9. (**A**,**D**), A549 cells were transfected synthetic miR-9 mimic (left), miR-9 inhibitor (right), or their relative control for 48 h. The total RNAs and the total protein were purified and subjected to qRT-PCR assay (**A**) and western blot anlaysis (**D**) respectively. The 105 kDa and 50 kDa blots of NFκB were cropped from the same gel and run under the same experimental conditions. Fold change of each treatment vs. control was calculated after quantification and presented under each blot. (**B**) schematic seed region sequences of miR-9 aligned with the FoxO1 3′-UTR wild type plamid and the mutant plasmid. (**C**) A549 cells were transfected with the miR-9 mimic or its control with FoxO1 3′-UTR wild type or mutant plasmids as indicated for 24 h. The Renilla plasmid was cotransfected for normalization. Then cells were harvested and subjected to fluorescent reporter assay. The Columns, means of three replicate determinations; bars, SD. *P < 0.05. The data are representatives of three independent experiments.

MiRNAs function through regulation of downstream targets. Accumulating evidences have shown that the target genes of miR-9 include NF-κB, FoxO1, CDX2 *et al.* in other types of cancers. However, for its targets in lung cancer is still unclear. Since miR-9 was identified as an oncogene in lung cancer, we suspected that its target genes were tumor repressors. We first detected the effects of miR-9 on FoxO1 expression. Figure 3A showed that transfection of miR-9 mimics or inhibitors had no effect on FoxO1 mRNA expression. However, the mRNAs of NF-κB, another direct target of miR-9, were negatively regulated. We further confirmed this finding by detecting FoxO1 mRNA expression with another three pairs of primers located in the different regions of FoxO1 mRNA. It showed that FoxO1 mRNA was not regulated by miR-9 (See supplementary information). Considering that miR-9 seed region did not completely match with the 3′-UTR region of FoxO1, we hypothesized that miR-9 may regulated FoxO1 protein levels through inhibition of mRNA translation but not mRNA degradation (Fig. 3B). We then constructed two plasmids inserted with the wild type 3′ -UTR of the FoxO1 containing the seed region recognized by miR-9 (FoxO1 3′-UTR WT), or the mutant 3′-UTR containing 4 nucleotides deletion in seed region (FoxO1 3′UTR mut) (Fig. 3B). The fluorescence reporter assay showed that miR-9 mimic cotransfection decreased the fluorescence intensity in cells transfected with the wild type 3′UTR of FoxO1 plasmid significantly compared with the miR-9 control cotransfection, while it could not decreased the fluorescentce intensity of cells with mutant 3′UTR plasmid transfection, suggesting that FoxO1 was a direct target of miR-9 (Fig. 3C). Further western blot analysis showed that miR-9 mimic decreased, while miR-9 inhibitor increased FoxO1 protein levels (Fig. 3D). As well, NF-κB was negatively regulated by miR-9. These results suggest that miR-9 negatively regulated FoxO1 translation through directly binding to its 3′-UTR.

**Figure 4:**
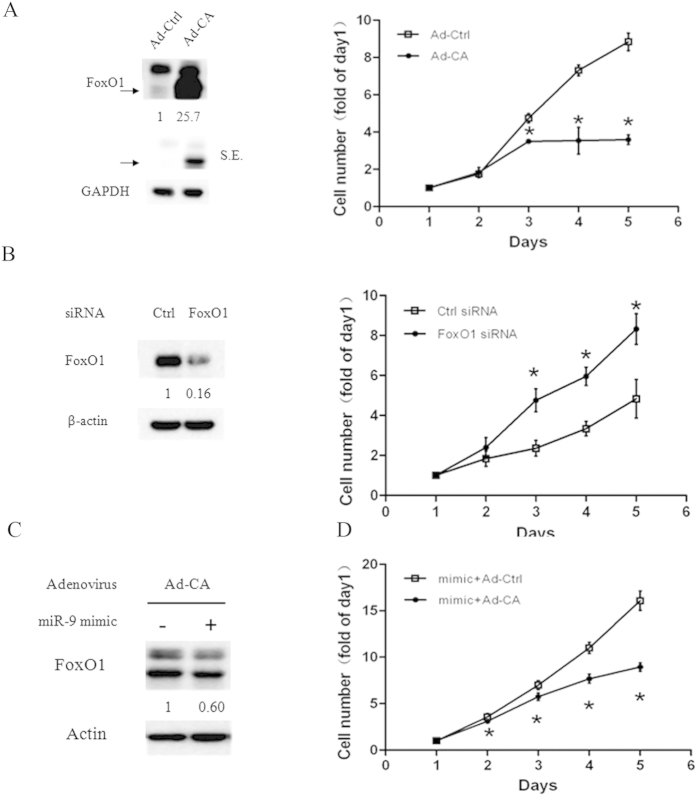
FoxO1 inhibited the growth of A549 cells and overexpression active FoxO1 partially reversed the effects of miR-9 on cell growth. (**A**) A549 cells in 6-well plates were infected with adenovirus encoding an active form of FoxO1 (Ad-CA) or its control (Ad-Ctrl) for 24 h (**A**), or transfected with FoxO1 siRNA or its control for 24 h (**B**) then subjected to western blot analysis (left) and a 5-day SRB assay (right). (**C**) A549 cells infected with Ad-CA and transfected with synthesized miR-9 mimic or its control simultaneously for 48 h, then subjected to western blot analysis. Fold change of each treatment vs. control was calculated after quantification and presented under each blot. S.E., shorter exposure. (**D**) A549 cells in 6-well plates were transfected with synthetic miR-9 and infected with Ad-CA or Ad-Ctrl simultaneously, then subjected to a 5-day SRB assay. Points, means of four replicate determinations; bars, SD. *P < 0.05. The data are representatives of three independent experiments.

Since both FoxO1 and NF-κB were regulated by miR-9 in NSCLCs, we next determined the critical role of FoxO1 in mediating the oncogenic function of miR-9 in NSCLCs. First, we determined the effect of manipulating FoxO1 expression on cell growth. A549 cells were infected with adenovirus which encode a constitutively active form (Ad-CA) of FoxO1, or the relative control adenovirus (Ad-control). Ad-CA significantly increased FoxO1 protein levels and inhibited cell growth (Fig. 4A). Accordingly, knockdown of FoxO1 expression using siRNA decreased FoxO1 protein levels and promoted cell growth significantly (Fig. 4B). These results confirm previous reports that FoxO1 is a tumor suppressor in NSCLCs.

Next, we determined whether overexpression of FoxO1 abrogated the pro-growth effect of miR-9. A549 cells were transfected with miR-9 mimic and infected with Ad-CA FoxO1 or its control. Western blot analysis showed that miR-9 decreased exogenous overexpressed FoxO1 protein by Ad-CA FoxO1 infection (Fig. 4C). And overexpression of FoxO1 partially inhibited miR-9 mimics transfection induced cell growth (Fig. 4D). These results suggest that FoxO1 is a downstream target of miR-9 in NSCLCs.

### Upregulation of FoxO1 by erlotinib is mediated partially through miR-9

**Figure 5:**
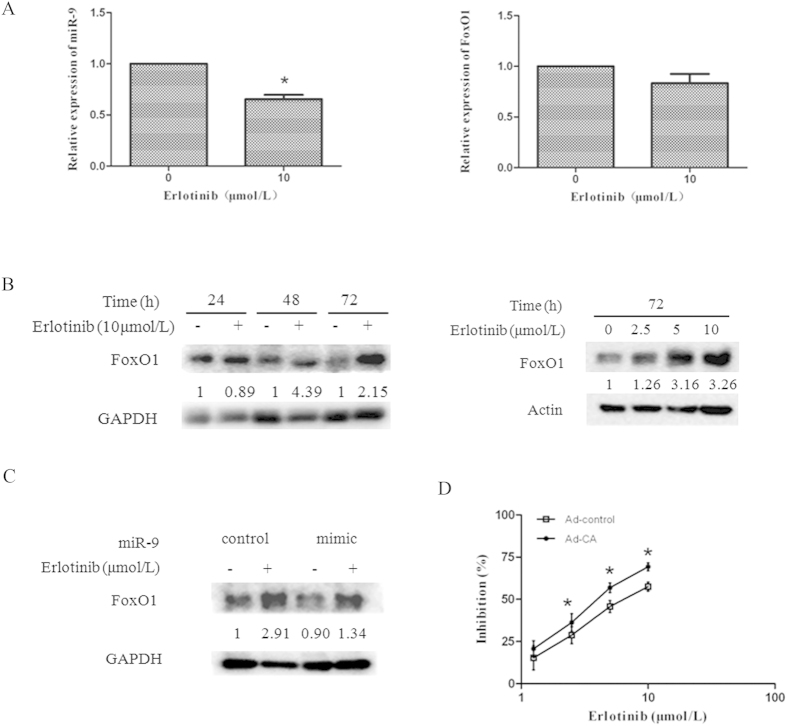
Erlotinib upregulated FoxO1 expression through downregulation of miR-9. (**A**) A549 cells were treated with or without erlotinib for 72 h, and subjected to qRT-PCR assay. (**B**) A549 cells were treated with erlotinib in different concentrations for different times as indicated and subjected to western blot analysis. (**C**) A549 cells were transfected with miR-9 mimic and its control for 24 h, then treated with 10 μmol/L erlotinib for another 48 h. The whole-cell lysates were purified and subjected to western blot analysis. Fold change of each treatment vs. control was calculated after quantification and presented under each blot. (**D**) A549 cells in 96-well plates were infected with adenovirus encoding an active form of FoxO1 (Ad-CA) or its control (Ad-Ctrl), then treated with or without erlotinib for 3 days and subjected to SRB assay. Columns, means of three replicate determinations; points, means of four replicate determinations; bars, SD. *P < 0.05. The data are representatives of three independent experiments.

Since erlotinib downregulated miR-9 expression and miR-9 negatively regulated FoxO1 protein levels, we further determined the effects of erlotinib on FoxO1 expression. Figure 5A showed that erlotinib decreased miR-9 expression without parallel increase of FoxO1 mRNA expression. However, erlotinib upregulated FoxO1 protein expression levels in both a time- and a dose-dependent manner (Fig. 5B). It suggests that erlotinib upregulates FoxO1 protein levels but not mRNAs, and the regulatory pattern is similar to miR-9.

To clarify the role of miR-9 in erlotinib-induced FoxO1 expression, we detected FoxO1 protein levels in A549 cells transfected with miR-9 mimic or it control, and then treated with or without erlotinib. Western blot analysis showed that erlotinib increased FoxO1 protein levels, whereas erlotinib and miR-9 mimic cotreatment decreased FoxO1 (Fig. 5C). It suggests that miR-9 play a critical role in erlotinib-induced FoxO1 expression.

In addition, we detected the effect of FoxO1 on erlotinib induced growth inhibition. Figure 5D showed that overexpression of an active form of FoxO1 enhanced the growth inhibitory effect of erlotinib on A549 cells compared with control. These results suggest that miR-9 regulated FoxO1 expression is a target of erlotinib in NSCLCs.

### Erlotinib downregulates miR-9 expression through activating the DNA methylation and subsequently suppressing the transcription of miR-9-1

**Figure 6:**
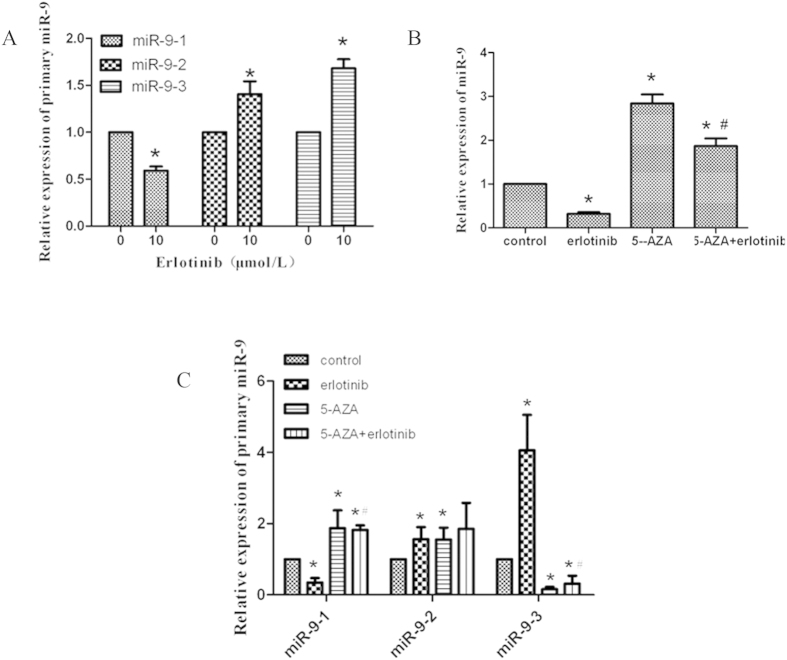
Erlotinib downregulated miR-9 expression mainly through enhancing DNA methylation mediated inhibition of miR-9-1 transcription. (**A**) qRT-PCR assay of A549 cells treated with or without erlotinib for 72 h. (**B**,**C**) A549 cells were treated with DMSO, 10 μmol/L erlotinib, 1 μmol/L 5-Azacytidine, or their combination for 72 h, and subjected to qRT-PCR assay. Columns, means of three replicate determinations; bars, SD. *P < 0.05, compared with control; ^#^*P* < 0.05, compared with erlotinib treatment. The data are representatives of three independent experiments.

As an EGFR inhibitor, the mechanism of erlotinib on decreasing miR-9 expression is unknown. Mature miR-9 comes from three miR-9 genes, located on Chromosomes 1, 5, and 15, named primary miR-9-1, -2, and -3, respectively. We first detected the primary miR-9 (pri-miR-9) expression after erlotinib treatment. Figure 6A showed that erlotinib decreased pri-miR-9-1, whereas increases pri-miR-9-2 and -3, suggesting the critical role of primary miR-9-1 in mediating erlotinib’s growth inhibitory effects. Furthermore, we found that DNA methyltransferase inhibitor 5-Azacytidine upregulated mature miR-9 (Fig. 6B) and pri-miR-9-1 significantly (Fig. 6C). And cotreatment with erlotinib and 5-Azacytidine abrogated mature miR-9 expression in parallel with pri-miR-9-1 expression when compared with erlotinib single treatment (Fig.6B,C). These results suggest that erlotinib downregulates miR-9 expression through suppressing the transcription of miR-9-1 and enhanced DNA methylation may be involved.

## Discussion

In this study, we defined the oncogenic effect of miR-9 in lung cancer. First, we detected increased miR-9 expression in 19/20 human NSCLC tissues compared with peripheral normal tissues. Second, overexpression of miR-9 transiently by transfection of exogenous synthesized miR-9, or permanently by establishing stable cell lines, promoted the growth of NSCLC cells. Even though transiently transfection of synthesized miR-9 inhibitors only slightly inhibited cell growth, the stable cell lines with downregulated miR-9 grew slowly than the control cells. These data suggest that miR-9 is oncogenic in NSCLCs. Aberrant miR-9 expression has been detected in several types of human cancer tissues. In gastric^14^, endometrial^11^, brain cancer^15^, and leukemia^9^, miR-9 is observed upregulated and oncogenic, whereas in cervical cancer^16^, colorectal cancer^17^, and ovarian cancer^18^ it is observed downregulated and anti-tumorigenic. Heller *et al.* reported that in non-small cell cancers, miR-9 expression was downregulated based on a genome-wide miRNA expression profiling. And DNA hypermethylation of primary miR-9-3 accounts for the downregulation of mature miR-9^19^. Our observations did not consistent with their findings, it may due to the tissue samples selected from the patients in different disease stages. We collected tissue samples from surgery patients, thereby most of whom were in early stage of the disease. However, our results that miR-9 promotes the growth of A549 cells suggest that miR-9 is oncogenic in NSCLCs. Recently, it is acknowledged that the same miRNA may play opposite roles in different cancer subtypes, in different stages of the diseases, or in different steps of chemotherapy, as our understanding of the miRNAs goes further^14^,^20^. Therefore, our findings suggest miR-9 as an oncogene at least in some of the lung cancer patients and possibly in early stages.

We also identified miR-9 as a new target of erlotinib, based on our findings that (1) erlotinib downregulated miR-9 expression in several NSCLC cells; (2) overexpression of miR-9 decreased the growth inhibitory effects of erlotinib. Since erlotinib mainly blocks EGFR, we examined the regulation of miR-9 by erlotinib in both A549 cells, containing wild-type EGFR, and HCC827 cells, containing an active mutant form of EGFR, our findings indicats that it is a common phenomenon. However, miR-9 overexpression only partially reversed erlotinib’s anti-growth effects, suggesting that downregulation of miR-9 expression is one of the mechanisms of erlotinib in addition to EGFR inhibition or is the consequence of EGFR inhibition. Myatt *et al.* reported that simultaneous overexpression of 2-8 miRNAs restored, whereas overexpression a single miRNA did not alter, the FoxO1 expression in endometrial cancer^11^. So, it is also possible that many miRNAs locate downstream of EGFR and are involved in erlotinib’s anticancer effects, thereby overexpression of a single miRNA is not enough to reverse erlotinib’s effects. Therefore, other microRNAs, in addition to miR-9 are worthy of further investigation.

Many miRNAs have been determined to be involved in EGFR signaling pathway and contribute to innate and acquired resistance of EGFR inhibitors. The let-7g is one of the best-studied miRNA that acts as a tumor suppress in lung cancer through negative regulation of K-Ras^21^. miR-7 showed anti-tumor effects through directly downregulation of EGFR in glioblastoma, breast cancer, and lung cancer^22^,^23^,^24^. For the specific miRNAs that regulated by EGFR inhibitors and the mechanisms have not been clarified. Many reports suggest that miR-9 expression is transcriptionally regulated by transcription factors such as c-Myc, by the DNA methylation of the promoter, and by the histone methylation or acetylation^19^,^25^,^26^. MiR-9 comes from three precursors named miR-9-1, -2, and -3, located in chromosome 1, 5, and 15, respectively^25^. In this study, we found that erlotinib-induced suppression of miR-9 was resulted from inhibition of miR-9-1 transcription, possibly involving a mechanism of enhanced DNA methylation. Senyuk *et al.* has reported that ecotropic viral integration site (EVI1) - induced suppression of miR-9 is determined by the hypermethylation of miR-9-3 promoter and involves induction of DNA-methyltransferase 3-β (DNMT3b)^9^. Considering that erlotinib inhibits two major surviving pathways in malignancy - PI3K/Akt and Ras/MAPK, it is possible that erlotinib activates DNA-methyltransferase through some molecules downstream of Akt or ERK. It is worthy of further investigation.

miRNAs functions through regulation of multiple genes expression at the posttranscriptional level. Several molecules that are critical in cancer development have been reported as direct downstream targets of miR-9. In gastric cancer, miR-9 targets CDX2 (caudal-related homeobox) to promote cell proliferation^14^. In ovarian cancer, miR-9 targets NF-κB to inhibit cell growth^18^. In leukemia, miR-9 targets FoxOs (FoxO1 and FoxO3) to promote cell differentiation^9^. In breast cancer, miR-9 targets E-cadherin to promote EMT (Epidermal-mesenchymal transition) and metastasis^25^. Furthermore, there exists much more predicted target genes of miR-9 by the software, such as TargetScan, Pictar. In this study, we identified the tumor suppressor - FoxO1 as a direct downstream target of miR-9 based on the luciferase reporter assay, which is in concomitant with that miR-9 is oncogenic in NSCLCs. We also revealed that miR-9 functions through suppressing FoxO1 translation but not mRNA degradation, based on our findings that miR-9 decreased protein levels, but not mRNA levels of FoxO1. To further confirm this finding, we detected NF-κB mRNA levels in the same samples, because it has been demonstrated that NF-κB was regulated by miR-9 at the mRNA level. The results that NF-κB mRNA was negatively regulated by miR-9 suggest that miR-9 expression was successful and it was functional in our experimental system. Moreover, three additional pairs of FoxO1 primer located in the different regions of FoxO1 mRNA were used to examine FoxO1 mRNA expression further. Results showed that FoxO1 mRNA was not regulated by miR-9 as well (See supplementary information). Additionally, only 7 bases in the 3′UTR region of FoxO1 matches the seed region of miR-9, this incomplete match indicates translational inhibition theoretically (Fig. 3B). In this study, since miR-9 is oncogenic, we predict that miR-9 - modulated tumor suppressor gene - FoxO1 plays a more critical role than miR-9 - modulated oncogene NF-κB under erlotinib treatment in NSCLCs.

In summary, the currently study identifies miR-9-regulated FoxO1 expression plays an important roles in promoting NSCLCs and suppression of this axis contributes to erlotinib’s anticancer efficacy. Moreover erlotinib downregulates miR-9 expression through suppress of primary miR-9-1 transcription. Our findings indicate a new mechanism of erlotinib and a new strategy to enhance EGFR-targeted cancer therapy through cotargeting miRNAs.

## Materials and Methods

### Reagents

Erlotinib (qE-4007) was purchased from LC Laboratories, and dissolved in DMSO at 20 mmol/L. 5-Azacytidine (A2385) was purchased from Sigma-Aldrich, and dissolved in PBS at 20 mmol/L. Stock solutions were at −20 °C and diluted just before use. Lipofectamine 2000 transfection reagent was purchased from Life Technologies Co. Invitrogen (11668-019). Antibodies FoxO1 (BS3573), actin (AP0064), and GAPDH (AP0063) were purchased from Bioworld Technology Inc. NFκB p105/p50 antibody (1559-1) was purchased from Epitomics, Inc. The synthetic miR-9 mimic, miR-9 inhibitor, and their relative control were purchased from Dharmacon.

### Cell lines and cell treatment

Human NSCLC cell lines A549, Calu-1, H157, H460, and HCC827 were purchased from the American Type Culture Collection (ATCC; Manassas, VA). Lentivirus encoding miR-9 mimic and its control (OE-miR-9/OE-Ctrl), or miR-9 inhibitor and its control (dMAN-miR-9/Cel-Ctrl) were purchased from Shenzhen Ongran Biotech Co,Ltd. A549 stable cell lines with miR-9 overexpression and its control (A549-OE-miR-9/A549-OE-Ctrl), or with miR-9 downregulation and its control (A549-dMAN-miR-9/A549-Cel-Ctrl) were established by infection of A549 cells with lentivirus aforementioned in 6-well plates for 48 h, selected with 2 μg/mL puromycin (P8833) purchased from Sigma for 14 days, and withdrew of puromycin for another 14 days as we previously described^27^. These cells were cultured in RPMI 1640 medium supplemented with 5% fetal bovine serum at 37 °C in a humidified atmosphere consisting of 5% CO_2_.

### Sulforhodamine B assay

Cells after transfection of the synthetic miR-9 or FoxO1 siRNA, or infection of the lentivirus or adenovirus for 24 h were reseeded to 96-well plates at 1,500 and cultured for 5 days, or at 2,000 cells/well and treated with erlotinib or its vehicle on the second day for 3 days. Cell number was estimated by the sulforhodamine B (SRB) assay and the growth inhibition was calculated as we previously described^28^.

### Quantitative real-time polymerase chain reaction (qRT-PCR)

Total-RNA from cells was extracted using Trizol reagent (1596-026) from Invitrogen Life Technologies, reverse transcription was conducted using RevertAid^TM^ Reverse Transcriptase (EP0441) from Thermo Fisher Scientific Inc., and quantitative PCR was conducted using FastStart Universal SYBR Green PCR Master mix (4913914001) from Roche, according to manufacturer’s procedure. Forward (F) and reverse (R) primers were used as follows: FoxO1, F: 5′-TGGACATGCTCAGCAGACATC-3′ and R: 5′-TTGGGTCAGGCGGTTCA-3′; NF-κB1, F: 5′-CCTGGATGACTCTTGGGAAA-3′ and R: 5′-TCAGCCAGCTGTTTCATGTC-3′, GAPDH, F, 5′-ATGGGGAAGGTGAAGGTCG-3′ and R, 5′-GGGGTCAT TGATGGCAACAATA-3′, and synthesized by Invitrogen^29^,^30^. TaqMan microRNA assay for miR-9 was purchased from Applied Biosystems Inc., and U6 small nuclear RNA (U6 snRNA) was used as normalization control. All real-time amplifications were measured in triplicates and performed with the ABI Prism 7300 sequence detection system (Applied Biosystems) as we previously described^28^,^31^. The fold-change of miR-9, FoxO1, and NF-κB1 was calculated using the 2^−ΔΔCT^ method.

### Western blot analysis

Whole-cell protein lysates were prepared and subjected for western blotting as described previously^32^. The chemiluminescent signal was collected and analyzed by Kodak Image Station. Index of Density (IOD) of each band = density × area. The value of IOD ratio (IOD ratio = IOD of FoxO1 or NF-κB / IOD of house-keeping gene) was calculated. The fold change (Fold change = IOD ratio of treatment / IOD ratio of control) was presented under each blot.

### Manipulating miR-9 expression transiently by synthetic miR-9 transfection or lentivirus infection

The synthetic miR-9 mimic, miR-9 inhibitor, and their relative control were purchased from Dharmacon. Cells seeded in 6-well plate at 5 × 10^5^ cells/well were transfected with synthetic miR-9 and its control using lipofectamine 2000, or infected with lentivirus aforementioned for 24 h. Then cells were reseeded to 96-well plates for a 5-day SRB assay, or 6-well plates for another 24 h for qRT-PCR assay and western blot analysis.

### Plamid construction and fluorescent reporert assay

The 3′-untranslated region (3′-UTR) of FoxO1 (170 nt) containing the predicted miR-9 binding site were synthesized by Vazyme Biotech Co., Ltd. The mutant 3′-UTR of FoxO1 were also synthesized except that 4 nucleotides in the seed region were deleted. These fragments were inserted into the pGL3-Basic (Promega) plasmid at Xbal enzyme digested site, and named as FoxO1 3′-UTR WT (wild type) or FoxO1 3′-UTR mut (mutant). All plasmids were verified by DNA sequencing. A549 cells were seeded in a 24-well plate and cotransfected with miR-9 mimic or its control with FoxO1 wild type or mutant plasmid for 24 h. Then cells were harvested following the instruction of the Dual-luciferase Reporter assay system purchased from Promega Co., and measured on fluorescence spectrometer (Promega GloMax 20/20 E5311) for the fluorescence intensity. The renilla vector was also cotransfected to normalize the transfection efficiency.

### Gene knockdown by small interfering RNA

Control (non-target) small interfering RNA (siRNA) was purchased from shanghai GenePharma Co. Ltd. FoxO1 siRNA that targets 5′- GCCCUGGCUCUCACAGCAATT-3′ was described previously and synthesized by shanghai GenePharma^33^. Cells were seeded to 6-well plate at 5 × 10^5^ cells/well and transfected with 100 nmol/L control or FoxO1 siRNA respectively for 24 h using lipofectamine 2000 (11668-019). Then cells were reseeded to 96-well plates for a 5-day SRB assay, or 6-well plates for another 24 h for western blot analysis.

### Gene overexpression by adenovirus

The adenovirus encoding an active form of FoxO1 (Ad-CA) and its control (Ad-Ctrl) carring GFP (green fluorescence protein) were purchased from Vazyme Biotech Co., Ltd. Cells in 6-well plates were infected with adenovirus for 24 h, then reseeded to 6-well plates for another 24 h for western blot anaylsis, or 96-well plates for a 5-day SRB assay, or 96-well plates for a 3-day SRB assay of erlotinib treatment. Synthetic miR-9 transfection and adenovirus infection were conducted simultaneouly when overexpression of both miR-9 and FoxO1 were required.

### Human tissue samples

Paired cancer tissues and peripheral normal tissues were collected from lung cancer patients accepted the surgery at the First Affiliated Hospital of Nanjing Medical University during 2010–2011. In 20 patients, 6 were adenocarcinoma and 14 were squamous carcinoma (mean age 65.3 years). Total-RNA was purified from these tissue samples stored at −80 °C and subjected to qRT-PCR assay. The study was approved by the Ethics Committee of Nanjing Medical University and informed consent was obtained from all the participating subjects. All experiments were performed in accordance with approved guidelines of the Nanjing Medical University.

### Statistical analysis

The statistical significance of differences in cell viability between different treatments was analyzed with two-sided unpaired Student’s t tests. Results were considered to be statistically significant at *P* < 0.05.

## Additional Information

**How to cite this article**: Chen, X. *et al.* Oncogenic miR-9 is a target of erlotinib in NSCLCs. *Sci. Rep.* **5**, 17031; doi: 10.1038/srep17031 (2015).

## Supplementary Material

Supplementary Information
