# Supplementary material for: Oncogenic miR-9 is a target of erlotinib in NSCLCs
Source: Sci Rep. 2015 Nov 23;5:17031. doi: 10.1038/srep17031 (PMC4655475; doi:10.1038/srep17031)
Supplement: Supplementary Information [file srep17031-s1.pdf]

Supplementary Information

Oncogenic miR-9 is a target of erlotinib in NSCLCs

Xi Chen[1], Lingjun Zhu[2], Zhuo Ma[1], Geng Sun[1], Xuan Luo[1], Min Li[1], Sulan Zhai[1], Ping Li[1], Xuerong Wang[1*]

[1]Department of Pharmacology, Nanjing Medical University, Nanjing, Jiangsu Province, China. 210029

[2]Department of Oncology, the First Affiliated Hospital of Nanjing Medical University, Nanjing, Jiangsu Province, China. 210029

[*]Corresponding: wangxrwn@hotmail.com

Supplementary Table

Table Primers of FoxO1 used in qRT-PCR assay

Primers	Forward	Reverse
Primer-1	5'-GCAGATCTACGAGTGGATGGTC-3'	5'-AAACTGTGATCCAGGGCTGTC-3'
Primer-2	5'-CCACACCTCGGGTATGAACC-3'	5'-GAGAAGGCCCATTCTGCCAT-3'
Primer-3	5'-GTATGAACCGCCTGACCCAA-3'	5'-CACTTGGGAGCTTCTCCTGG-3'

Note: Primer-1 was described previously [Reference: Chia-Chen Kuo, Shih-Chang Lin. Altered FOXO1 Transcript Levels in Peripheral Blood Mononuclear Cells of Systemic Lupus Erythematosus and Rheumatoid Arthritis Patients. Mol Med. 2007; 13 (11-12): 561-566]. Primer-2 and 3 were designed by software Primer5.

Supplementary Figure

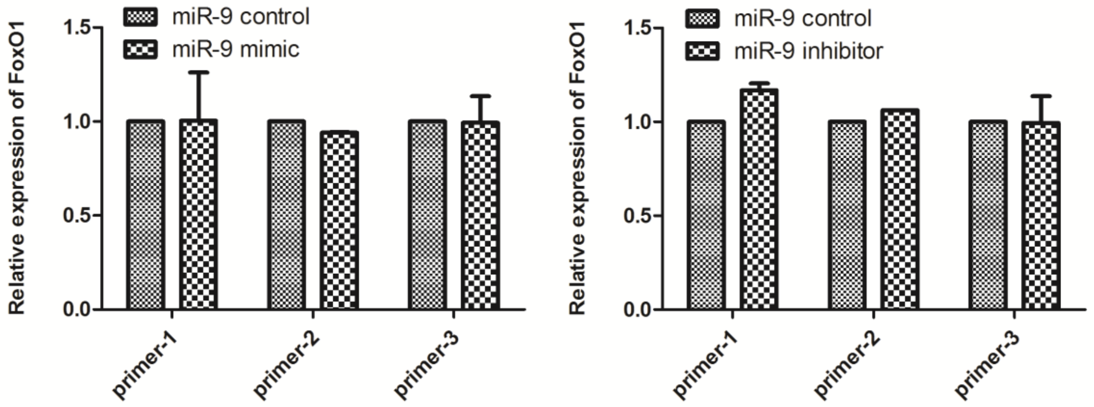

Figure: FoxO1 mRNA expression was not regulated by miR-9. A549 cells were transfected with synthetic miR-9 mimic (left), miR-9 inhibitor (right), or their relative control for 48h. The total RNAs were prepared and subjected to qRT-PCR assay. GAPDH expression was used as normalization control. The Columns, means of three replicate determinations; bars, SD.
